# A Letter to the Secretary of State for the Home Department, on Anatomy

**Published:** 1829-04-01

**Authors:** 


### A Letter to the Right Hon. the Secretary of State for the Home Department, on Anatomy, &c. By *G. J. Guthrie*, F.R.S. Professor of Anatomy and Surgery to the Royal College of Surgeons, &c. &c. Octavo, stitched, pp. 37. Sams, London, 1829. Price One Shilling.

We hope and trust that the difficulties and even dangers which have long beset the study and the teaching of anatomy in this country are fast disappearing, together with most of those popular prejudices, lamentable, though honest, and not to be scoffed at, by which they were engendered. We believe that a better æra is dawning on our science, and that soon a parliamentary enactment will allow us to proceed unmolested in our pursuit, and—

All the clouds that lower'd o'er our house,
In the deep bosom of the ocean bury.

The scenes that have lately been witnessed in Edinburgh, and which have raised against a gentleman teaching anatomy in that city, a feeling almost as strong, and violence almost as great, as a former Edinburgh mob displayed against the unfortunate Captain Porteus, though afflicting to the profession, disgraceful to human nature, and horrible in every point of view, will yet, we are convinced, be attended in the end with beneficial results. They will display in their true colours the infamous miscreants called into action by the present laws against the open cultivation of anatomy, and shew that an over-regard for the dead has occasioned, *is* occasioning, and, we very much fear, will still occasion, the MURDER of the living.

"Murder most foul, as at the best it is,
But this most foul, strange, and unnatural."

Whilst all, however, fully acknowledge the evil, many opinions exist as to the nature of the remedy our state-physicians should apply. The Parliamentary Committee, and the Chairman, Mr. Warburton, in particular, are above all praise for the pains which they have taken in collecting evidence, and their talents in embodying that evidence into the recommendations to the Legislature, which form the pith of their REPORT. At the same time, it is not quite clear that all which might be said, has been said upon the subject, and on that account we have been led to take notice of the pamphlet, published within these few days by Mr. Guthrie. Without fully agreeing in all that that gentleman has advanced, we think that his remarks are deserving of attention, particularly in the quarter from which a bill on the subject is to emanate. The honourable gentleman, Mr. Warburton, who already has displayed such zeal and candour, will, we feel assured, glean all the information he can, before he ventures on the final step. Our space is so limited, that all we can do at present is, to place before our readers the plan and resolutions proposed by the distinguished author of the pamphlet, without indulging in any observations of our own.

"The following plan is proposed under this system, and I would recommend the supply of dead bodies for public dissection to be derived from the following sources:—

"1. All persons hanged, or otherwise executed, and for all offences whatsoever.

"2. All persons who die under sentence for criminal offences, whether in the hulks, gaol, penitentiary, or elsewhere.

"3. All persons who die in temporary or floating hospitals, in gaol, penitentiary, or other place of detention, or prison, from whatever cause they have been placed there, and who have no friends to bury them.

"4. All persons found dead, from whatever causes, in highways, canals, or otherwise, and who, having no friends to bury them, are sent to bone-houses for interment, at the expense of the parish or county.

"5, *and lastly*. The poor who die in workhouses, having no friends to bury them, having expressed no wish on the subject, and having no respectable or decent relatives to express it for them, either before or after death.

"It is not proposed to interfere by regulations with the bodies of those who die without friends in regularly established hospitals; it being presumed that the surgeons of those institutions will properly apply them in the instruction of the students committed to their charge. In other words, it is not intended that the public schools of anatomy shall interfere with the private or public instruction delivered by surgeons in their own hospitals.

"The means of supply being furnished, the following regulations are proposed, to ensure a fair and regular distribution, which must be enforced, in one way or other, by legal enactment. It being understood that there are no laws on the subject to repeal, save that one, or part of one, which directs murderers to be hanged until dead, 'and their bodies to be given over for dissection,' and for the reception of which bodies the College of Surgeons is bound, by their charter, to find a proper place, which is at present in the vicinity of Newgate.

"*Laws proposed to be enacted.*—1. Punishing all persons actually engaged in exhumating or stealing a dead body, or of selling it without authority, and who can be proved to have been so engaged after this Session of Parliament. For the first offence, six months to hard labour, and to find two securities, in fifty pounds each, for future good behaviour; to be kept to hard labour until procured. For the second offence, double the punishment. Medical or other persons knowingly receiving such dead bodies, three months to the treadmill, and a fine of one hundred pounds; to be kept to hard labour until paid.

"2. Rendering the practice of dissection, and the possessing of dead bodies, legal; and protecting the persons so employed, and their property, by the same laws as protect persons and property generally.

"3. Directing the five sources of supply of dead bodies, as at pages 30 and 31.

"4. Declaring it to be illegal to require or to take during life, in any hospital, work-house, or other place for the reception of sick, or poor people, securities in money or otherwise for the burial of such persons. Penalty, twenty pounds.

"5. Declaring it legal, and directing all treasurers, governors, trustees, or others in authority, in hospitals or other places; and all vestries, church-wardens, overseers of the poor, and others in authority in the parishes, to give over for dissection to the College of Surgeons, or persons appointed by them, the bodies of all persons who have died, under their care or charge, without the means of burying them, and who have no relatives or persons previously known to have been friends, who are willing to do it; and all other bodies in their charge which come within the meaning of classes four and five, of the means of supply indicated, pages 30 and 31.

"6. Appointing the Royal College of Surgeons of London, by their secretary, or other person nominated by them, the proper authority or authorities, to whose order the bodies are to be delivered.

"7. The Royal College of Surgeons to report quarterly to the Secretary of State for the Home Department, on every point connected with this subject.

"8. The funeral service to be read over all bodies (unless forbidden by law,) before delivery for dissection.

"9. Legalising the sale of a dead body by the friends of the deceased, after it has been viewed in the usual manner by the parish or other authorities

"10. The Council of the Royal College of Surgeons, in making regulations for the proper distribution of the bodies placed at their disposal, to find a proper cemetery in various parish church-yards for the interment of remains after dissection; and the Council of the College to be authorised to make such charge for each body as may be considered proper; subject to the approval of the Secretary of State for the Home Department.

"11. All minor regulations of arrangement and detail made by the Council of the Royal College of Surgeons, and approved by the Secretary of State for the Home Department, to be binding on the different persons concerned. Penalty, twenty pounds.

"12. Every dispute which may occur, and every offence to which a penalty is attached, to be settled by information laid in the usual manner, before any three police magistrates of the division in which the offence has been committed; and whose decision shall be final.

"In order to enable all parties to act with precision, and a due regard to decorum, the following minor arrangements are proposed, under the authority of the Secretary of State, to be varied from time to time, by his sanction.

"The Council of the Royal College of Surgeons, having the collection and distribution of all dead bodies intended for dissection, directs.

"1. An establishment of men, four or eight in number, to be ready for service every evening in the winter season from six to ten o'clock, and to proceed as directed with a shell (in a manner similar to that at present adopted by undertakers), to the spot where the body is to be found.

"An establishment of one or two plain hearses, with two horses, a driver, and an attendant, in black (like an undertaker's party), to be ready to go to greater distances.

"3. The secretary, or proper officer appointed by the college, gives an order for the delivery of the body, which will be the receipt to the person who delivers it.

"4. The servant of the college who receives the body, delivers it again, according to an order received to that effect from the secretary; and the anatomist or gentleman who receives the body from him, gives an acknowledgment, signed by himself or his assistant." 35.

